# Enhancing soil resilience and crop physiology with biochar application for mitigating drought stress in durum wheat (*Triticumdurum*)

**DOI:** 10.1016/j.heliyon.2023.e22909

**Published:** 2023-11-28

**Authors:** Sonia Boudjabi, Nawal Ababsa, Haroun Chenchouni

**Affiliations:** a Department of Nature and Life Sciences, Faculty of Exact Sciences and Nature and Life Sciences, University of Tebessa, 12002 Tebessa, Algeria; b Laboratory “*Water and Environment*”, Faculty of Exact Sciences and Nature and Life Sciences, University of Tebessa, 12002 Tebessa, Algeria; c Laboratory of Natural Resources and Management of Sensitive Environments ‘RNAMS’, University of Oum-El-Bouaghi, 04000 Oum-El-Bouaghi, Algeria; d Department of Ecology and Environment, Faculty of Nature and Life Sciences, University of Khenchela, 40016 El-Hamma, Khenchela, Algeria; e Department of Forest Management, Higher National School of Forests, 40000 Khenchela, Algeria; f Laboratory of Algerian Forests and Climate Change, Higher National School of Forests, 40000 Khenchela, Algeria

**Keywords:** Above-ground biomass, Biochar, Water deficit stress, Plant physiology, Crop production, Soil fertility, Durum wheat, Drought impacts, Soil physicochemical properties, Organic matter enrichment, Environmental sustainability

## Abstract

The use of biochar has recently garnered significant attention as an agricultural management technique highly endorsed by the scientific community. Biochar, owing to its high carbon content, contributes to increased organic matter storage in the soil, consequently enhancing crop growth. This study aimed to elucidate changes in physicochemical soil fertility and durum wheat (*Triticum durum*) var. Vitron production under the influence of three biochar doses (0 g/kg, 5 g/kg, and 15 g/kg of soil) in combination with varying levels of drought stress (100 %, 80 %, 40 %, and 20 % of field capacity 'FC'). Notably, we observed a substantial increase in all physicochemical soil parameters, except for active calcium carbonate equivalent (ACCE), which displayed lower values (8.78 ± 1.43 %) in soils treated with biochar compared to control soil (15.69 ± 4.03 %). The biochar dose of 5 g/kg yielded the highest moisture content (8.81 %) and pH value (7.83). However, the highest organic matter content (4.89 ± 0.17 %) and total calcium carbonate equivalent ‘TCCE’ (3.67 ± 0.48 %) were observed with the dose 15 g/kg. Nevertheless, regarding plant growth, no improvements were observed in terms of height and above-ground biomass (AGB). Conversely, leaf surface area exhibited significant changes with biochar application, along with an increase in chlorophyll pigment content. On the other hand, drought stress significantly hindered plant height, AGB, and leaf water reserves, resulting in values of 13.48 ± 1.60 cm, 1.57 ± 0.31g/plant, and 41.79 ± 1.67 %, respectively. The interaction between biochar and water stress appeared to mitigate and limit the impact of stress. Notably, an enhancement in organic matter storage and soil water reserves was observed. For example, the moisture content in the control soil was 6.95 %, while it increased to 12.76 % for 15g biochar/kg and 80 % FC. A similar trend was observed for organic matter, TCCE, and electrical conductivity. This effect positively influenced chlorophyll *a and b* content, as well as leaf water content. However, when stress was combined with biochar amendment, plant height and AGB decreased. The addition of biochar improved soil fertility and physiological parameters of wheat plants. Nevertheless, when combined with water stress, especially in cases of reduced water reserves, productivity did not witness any significant improvements.

## Highlights

• Biochar amendment increased soil alkalinity but alleviate negative effects of water stress.
• Application of high biochar dose slightly decreases soil pH.
• Soil fertility parameters improved better with 5 g/kg dose of biochar than 15 g/kg.
• Biochar improved plant RWC, chlorophyll *a* and *b* under drought and water stress.
• In the short-term biochar did not improve significantly crop production.

## List of abbreviations

ACCE: active calcium carbonate (CaCO_3_) equivalent
AGB: above-ground biomass
B (0, 1, 2): biochar (doses)
Chl a: chlorophyll *a*
Chl b: chlorophyll *b*
*df*: degrees of freedom
DM: dry matter
DS (0, 1, 2, 3): drought stress (levels)
EC: electrical conductivity
FC: Field capacity
GLMM: generalized linear mixed-effects model
LSA: leaf surface area
pH: potential hydrogen
RDA: redundancy analysis
RWC: relative water content
SD: standard deviation
SM: soil moisture
SOM: Soil organic matter
TCCE: total calcium carbonate (CaCO_3_) equivalent

## 1 Introduction

Sustaining soil quality and nutritional status in arid and semi-arid regions presents a major challenge for many developing countries [1]. These regions grapple with infertile and nutrient-poor soils, posing a significant obstacle to crop development, particularly cereal production, the predominant crop in these areas [2]. Soil fertility loss primarily stems from the excessive exploitation of agricultural land, exacerbating issues related to soil degradation and land desertification [3]. However, it is essential to acknowledge that the harsh climate characteristic of arid and semi-arid regions stands as the primary factor limiting grain productivity. Indeed, various crops face substantial abiotic stress, notably drought stress (DS), which adversely affects their growth and yield [4]. During drought conditions, the soil's capacity to retain water diminishes, leading to osmotic stress that restricts the flow of water and nutrients to plants. Elevated temperatures further intensify evaporation, depleting the soil's water reserves. This drought stress, which impacts plants during their early and critical developmental stages, disrupts their growth [5,6].

Environmental stresses including droughts result in diminished nutritional quality of grains and, consequently, reduced yields of cereal crops, especially for durum wheat. Durum wheat (*Triticum durum* Desf.) stands as the most essential crop in North Africa, serving as the primary staple food in the region. In Algeria, this cereal features prominently in various dishes [7]. The cultivation of durum wheat in arid and semi-arid regions consistently faces recurring water stress challenges. Confronted with this situation, the adoption of effective solutions to rectify soil fertility becomes an urgent necessity. Organic fertilization emerges as the sole viable solution in this context. Organic fertilizers, rich in carbon content, enhance water retention in the soil [8]. The influence of agronomic techniques such as fertilization, pH adjustments, plowing, irrigation, and organic amendments on soil multifunctionality is a subject of global interest among scientists [9]. Among the biofertilizers increasingly adopted by farmers, biochar holds a prominent place.

Biochar is a beneficial component that plays a pivotal role in enhancing soil properties and functions [10]. Numerous studies [11,12] have documented an increase in soil water retention attributed to this component. According to the findings presented by Ref. [13], biochar enhances both the morphological and physiological attributes of basil plants, while also boosting soil enzymatic activity, ultimately promoting basil growth and yield. This bio-solid facilitates and enhances ion transfer capacity in the soil solution [14]. Paneque et al. [15] have reported that biochar amendments reduce the uptake of heavy metals by plants. In an experiment conducted by Ref. [16], wheat plants were subjected to both cadmium stress and drought in the presence of biochar. The results indicate that biochar improves plant morphological and physiological parameters, mitigates oxidative stress, lowers cadmium levels, and enhances antioxidant enzyme activity.

The utilization of biochar has demonstrated its ability to enhance the physiological and morphological characteristics of ginger [17]. Numerous research studies have likewise highlighted physiological improvements in plants subjected to stress conditions and treated with biochar [18]. In their study, Haider et al. [1] determined that the application of biochar (at a rate of 37.18 g/kg) can effectively enhance wheat grain yield by mitigating the detrimental impacts of DS. Given these encouraging findings, the interest in using biochar as a biofertilizer continues to grow.

The primary objective of this investigation is to assess the response of a cereal plant, specifically Durum Wheat, and to analyze the changes in fertility parameters in organic matter-deficient soil (Cambisol) when exposed to increasing doses of biochar in combination with varying levels of DS. Our hypothesis posits that the incorporation of biochar, in conjunction with DS, will enhance the physiological and productivity aspects of durum wheat. This enhancement is expected to exhibit a linear relationship with the dosage of biochar used. Additionally, biochar is anticipated to boost soil nutrient content and ameliorate the impact of stress by enhancing water retention in the soil [19]. This study distinguishes itself from prior research in several key ways. Firstly, it focuses on a hot semi-arid region in North Africa (Tebessa, NE Algeria) characterized by consistent water stress, replicating these conditions under controlled settings to meticulously explore the impact of biochar and varying drought stress levels on wheat plant development and soil fertility. Additionally, unlike some earlier studies that concentrated solely on biochar's impact on soil properties, this research delves into the intricate interplay between biochar doses and drought stress levels. It provides novel insights into how these factors interact, influencing both plant development and soil characteristics. Furthermore, this study bridges the gap by considering the specific challenges posed by soil deficiency in organic matter and high calcium carbonate content (haplic calcisols), thus offering valuable guidance for addressing agricultural issues in regions with similar soil characteristics. Tthis research contributes to the literature by advancing our understanding of biochar's role in improving soil fertility and plant resilience under water-stressed conditions in hot semi-arid regions where these challenges are prevalent, ultimately offering practical solutions to enhance agricultural sustainability.

## 2 Materials and methods

### 2.1 Study design

The objective of this study is to investigate the changes in soil fertility resulting from the application of increasing doses of biochar and to assess the impact of this bio-solid on the physiology and yield of durum wheat (*Triticum durum*), specifically the Vitron variety. The experiment was conducted in a greenhouse during the cultivation season 2021–2022 at the Faculty of Exact and Nature Sciences, University of Tebessa, situated in a semi-arid region in the Northeast of Algeria.

The experimental setup consisted of 36 pots, each with a capacity of 3 kg, filled with soil sourced from the local region. These pots were organized into three distinct blocks. The first block served as the control group, devoid of any biochar application, while the second block received a biochar dosage of 5g per kilogram of soil. The third block was treated with a higher biochar dose of 15 g/kg soil. Within each block, the pots were further subdivided into three replicates, and for each level of biochar used, there were four different irrigation levels applied, resulting in a total of 36 pots for the experiment.

Following the acquisition of wheat grains from the OAIC of Tebessa, we initiated the sowing process on December 12, 2021. In each pot, seven grains were carefully planted, and after watering, the pots were transferred to the greenhouse. The biochar used in this experiment was sourced from a local vendor. It was produced from woody branches and trunks of Allepo pine (*Pinus halepensis* Mill.), which were thoroughly crushed and transformed into a fine powder with a diameter of approximately 2 mm. This biochar was then added to the pots on the same date as the sowing.

The irrigation schedule was closely monitored, taking into consideration the soil's field capacity (FC), which had been determined at the outset of the experiment. The application of DS, in accordance with the aforementioned levels, was initiated after the formation of the fourth leaf on the plants. Prior to commencing the experiment, both the soil and the biochar underwent a comprehensive chemical analysis to determine their chemical and physical characteristics, as outlined in Table 1. The analysis revealed that the soil exhibited a deficiency in organic matter and high accumulation of calcium carbonates, a common characteristic of haplic calcisols (CLha) in the steppe region situated in northeastern of Algeria [3,20].

**Table 1:** Physicochemical characteristics of the soil and biochar used in the current experimental study.

Measured parameters	Unit	Soil	Biochar
Potential hydrogen (pH)		7.17 ± 0.30	8.96 ± 0.73
Electrical conductivity (EC)	μS/cm	583 ± 116	1320 ± 192
Nitrates (NO_3_^−^)	mg/g	3.45	8.32
Active CaCO_3_ (ACCE)	%	2.54 ± 0.20	4.66 ± 0.45
Total CaCO_3_ (TCCE)	%	13.74 ± 2.17	14.34 ± 1.87
Organic carbon (OC)	%	0.90	7.88 ± 0.25

### 2.2 Soil analysis

#### 2.2.1 Physicochemical of soil fertility variables

The pH measurement was conducted by creating a soil filtrate through the agitation of 20g of soil in 50 ml of distilled water for a duration of 30 min [21]. Similarly, electrical conductivity was determined using a conductivity meter, with readings taken from a soil filtrate prepared by shaking 10g of soil in 50 ml of water for a period of 2 h [22]. This parameter was measured using a multiparameter meter (model: Hanna HI9829).

Regarding soil moisture (SM) content, the method employed involved taking the initial fresh weight of 100g of wet soil and subsequently subjecting it to drying in an oven at 105 °C until a constant dry weight was achieved. The difference in weight before and after drying was used to calculate SM content using the following formula: Humidity (%) = (fresh weight - dry weight)/dry weight × 100.

#### 2.2.2 Chemical fertility variables

The active calcium carbonate equivalent (ACCE) was extracted in an oxalate solution by shaking (2h), the filtrate heated in the presence of sulphuric acid at a temperature of 60 °C, then titrated with potassium permanganate (0.2 N) until a persistent pink color is obtained corresponding to a volume V' (mL), the steps are repeated for a control sample without soil whose titration corresponds to the volume V [23]. The content of active calcium carbonate equivalent (ACCE) was calculated as follows: ACCE (%) = V–V' × 0.05 × 100/0.2.

For the total calcium carbonate equivalent (TCCE), this parameter was deduced by putting 2g of soil in a beaker containing HCl diluted to 1/3 whose weight is (P1). After the release of CO_2_, the beaker is weighed again to obtain the weight P2. The volume of CO_2_ released (P2–P1) was used to deduce the TCCE content according to the formula: TCCE (%) = weight of CO_2_ released × 2.274 × 100/weight of soil [23].

The determination of total carbon content followed the Walkley-Black method [24]. In this process, soil organic matter was oxidized by potassium dichromate under acidic conditions. Any excess dichromate not reduced by organic carbon was subsequently titrated using a reducing solution containing Mohr's salts (ferrous sulfate). This titration was carried out in the presence of a colored indicator, diphenylamine, which produces a dark green coloration. The carbon content (C%) was calculated by taking the difference between the titration volume of the control sample without soil (X) and the sample containing soil (X′) and then dividing this difference by the weight of the test sample (Ws), followed by multiplication by 100, as per the formula: C% = (X - X′) × 0.6/Ws × 100.

### 2.3 Plant measurements

#### 2.3.1 Morphological parameters

The leaf area at the bolting stage was calculated by multiplying the length and width of the leaves and then multiplying the result by 0.75 [25]. To measure the height of the plants at the bolting stage, a long ruler was used. The height measurement was taken from the base of each selected plant to the highest point of the plant.

#### 2.3.2 Growth and physiological traits

At the maturity stage, all the plants (six plants in total) were harvested and divided into three replicates, with each replicate containing two plants. After weighing the plant material using a precision balance, we obtained the fresh aerial biomass. All the leaves that were used to estimate height and leaf area at the bolting stage were also collected for the determination of relative water content and chlorophyll content. For the extraction of chlorophyll *a* and *b*, we followed the Burnison method [26]. A maceration was carried out using 100 mg of fresh leaf material (FW) in test tubes containing a volume (V) of 10 mL of a mixture of acetone and ethanol in a respective proportion of 75 % acetone and 25 % ethanol. These tubes were then kept in the dark for 48 h to prevent chlorophyll oxidation due to light exposure. The optical densities were measured using a spectrophotometer at two specific wavelengths (WL): 645 nm for chlorophyll *a* and 663 nm for chlorophyll *b*. The content of these two pigments, chlorophyll *a and b*, was quantified using the following formulas:

Chla(μg/100mgFM)=12.7×WL(663)–2.59×DO(645)×V/(1000×FW).

Chlb(μg/100mgFM)=22.9×WL(645)–4.68×DO(663)×V/(1000×FW).

To determine the relative water content (RWC) of the leaves, the following technique was employed: (i) the fresh weight (FW) of three leaves from each pot was measured; (ii) these leaves were then immersed in distilled water for a period of 24 h to reach full turgor, and their weight at turgor (TW) was recorded; and (iii) subsequently, the leaves were dried to obtain their dry weight (DW). The RWC was calculated using the following formula:

RWC(%)=[(FW−DW)/(TW−DW)]×100

### 2.4 Statistical analysis

The data on soil physicochemical parameters and morpho-physiological traits of wheat plants were analyzed using various statistical methods. Descriptive statistics, including mean, standard deviation, and range, were used to summarize the observed values for different biochar doses and DS levels. The variations in these parameters among the combinations of biochar doses and DS levels were examined using generalized linear mixed-effects models (GLMM), with biochar doses, DS levels, and their interaction as fixed effects, and the experimental blocks as a random effect. To compare the means among biochar doses, DS levels, and their combinations for each soil parameter and plant trait, Tukey post-hoc tests (HSD) were conducted. The significance threshold was set at *p* < 0.05. All statistical analyses were performed using R version 4.3.0 [27]. Pearson correlation tests were used to assess the relationships between soil parameters and between plant traits measured under different biochar treatments and DS levels. The resulting correlation matrix for soil parameters was visualized using an interactive plot created with the R package "corrplot" version 0.92. Furthermore, a redundancy analysis (RDA) was conducted to investigate the overall relationships and correlations between all soil physicochemical parameters and plant morpho-physiological traits under different DS levels and biochar treatments. The RDA triplot was scaled and visualized using the "biplot" library version 0.92, and boxplots were generated using the "ggplot2" package.

## 3 Results

### 3.1 Soil properties

#### 3.1.1 Soil moisture

Soil moisture (SM) significantly increased (GLMM: *χ*^2^ = 8.03, *p* = 0.018) due to the influence of biochar, reaching its highest value at 8.81 ± 1.76 with the B1 dose, closely approaching the maximum content of 12.76 % observed throughout the entire experiment (Table 2, Table 3). The results can be categorized into three groups: B1 > B2 > B0, highlighting the varying impact of different biochar doses. The introduction of DS led to a consistent and significant linear decrease (*χ*^2^ = 8.69, *p* = 0.034) in SM content (Table 3). Average values ranged from 8.65 ± 1.57 % for the 100 % water level to 7.51 ± 1.92 % for DS3 (Table 2). Furthermore, the interaction between DS and biochar (DS × biochar) had a positive and significant effect (*χ*^2^ = 19.24, *p* = 0.004) on soil water content (Table 3). The highest soil water content (12.76 %) was observed at the 80 % water level with the B1 dose, while the lowest average moisture content (3.58 %) was recorded in the control soil without biochar, also at the same 80 % water level (Table 2).

**Table 2:** Mean (±SD) and range[minimum‒maximum] values of morphologic parameters measured on durum wheat plants grown in soil amended with biochar under four drought stress (DS) levels (100 %, 80 %, 40 %, 20 % FC). Superscript letters are the results of Tukey post-hoc tests (HSD) with lowercase letters for the interaction ‘Biochar × DS’, Greek letters for biochar doses, and uppercase letters for SS levels. Different letters indicate statistically significant differences (*p* < 0.05).

Variables	Biochar amendment doses			All biochar doses combined
Drought stress (DS) levels	B0 = 0 g/kg	B1 = 5 g/kg	B2 = 15 g/kg	
Soil moisture (%)				
DS0 = 100 % FC	8.35 ± 1.17 ^abc^ [6.95–10.19]	8.19 ± 1.69 ^abc^ [5.65–10.75]	9.41 ± 1.66 ^ab^ [6.6–11.41]	8.65 ± 1.57^A^ [5.65–11.41]
DS1 = 80 % FC	6.87 ± 2.1 ^bc^ [3.58–8.51]	9.75 ± 1.51^a^ [7.42–12.76]	9.09 ± 2.16 ^abc^ [6.04–11.98]	8.57 ± 2.25^A^ [3.58–12.76]
DS2 = 40 % FC	9.27 ± 1.83 ^ab^ [6.61–11.04]	8.53 ± 2.16 ^abc^ [5.49–11.46]	8.24 ± 1.42 ^abc^ [6.46–10.8]	8.68 ± 1.81^A^ [5.49–11.46]
DS3 = 20 % FC	6.34 ± 2.32^c^ [3.7–10.75]	8.78 ± 1.49 ^abc^ [7.02–11.35]	7.39 ± 1.01 ^abc^ [6.11–9.11]	7.51 ± 1.92^A^ [3.7–11.35]
All drought stress levels combined	7.71 ± 2.17 ^β^ [3.58–11.04]	8.81 ± 1.76 ^α^ [5.49–12.76]	8.53 ± 1.74 ^αβ^ [6.04–11.98]	
pH				
DS0 = 100 % FC	7.29 ± 0.4^c^ [6.44–7.78]	7.79 ± 0.15^a^ [7.56–7.98]	7.71 ± 0.16 ^ab^ [7.43–7.98]	7.59 ± 0.34^A^ [6.44–7.98]
DS1 = 80 % FC	7.48 ± 0.24 ^abc^ [6.98–7.82]	7.83 ± 0.1^a^ [7.65–8.01]	7.76 ± 0.06^a^ [7.68–7.85]	7.69 ± 0.22^A^ [6.98–8.01]
DS2 = 40 % FC	7.34 ± 0.39 ^bc^ [6.64–7.67]	7.85 ± 0.14^a^ [7.68–8.16]	7.62 ± 0.11 ^abc^ [7.42–7.75]	7.6 ± 0.32^A^ [6.64–8.16]
DS3 = 20 % FC	7.51 ± 0.52 ^abc^ [6.79–8.03]	7.86 ± 0.21^a^ [7.55–8.18]	7.63 ± 0.17 ^abc^ [7.39–7.85]	7.66 ± 0.36^A^ [6.79–8.18]
All drought stress levels combined	7.4 ± 0.39 ^γ^ [6.44–8.03]	7.83 ± 0.15 ^α^ [7.55–8.18]	7.68 ± 0.14 ^β^ [7.39–7.98]	
**Electrical conductivity (μS/cm)**				
DS0 = 100 % FC	1485 ± 475 ^ab^ [872−1964]	1112 ± 241 ^abc^ [759−1385]	1219 ± 244 ^abc^ [856−1520]	1272 ± 362 ^AB^ [759−1964]
DS1 = 80 % FC	1499 ± 165 ^ab^ [1185–1701]	1537 ± 424 ^ab^ [995−1998]	1260 ± 259 ^abc^ [976−1610]	1432 ± 316^A^ [976−1998]
DS2 = 40 % FC	1065 ± 241 ^abc^ [729−1333]	1586 ± 485^a^ [926−1924]	1433 ± 330 ^ab^ [985−1727]	1361 ± 416^A^ [729−1924]
DS3 = 20 % FC	748 ± 114^c^ [625−985]	1059 ± 415 ^bc^ [546−1520]	1565 ± 339 ^ab^ [1035–1945]	1124 ± 458 ^B^ [546−1945]
All drought stress levels combined	1199 ± 419 ^α^ [625−1964]	1323 ± 454 ^α^ [546−1998]	1369 ± 316 ^α^ [856−1945]	
**Soil organic matter (%)**				
DS0 = 100 % FC	3.01 ± 0.43 ^d^ [1.88–3.21]	4.09 ± 0.21 ^b^ [3.84–4.48]	4.96 ± 0.16^a^ [4.71–5.1]	4.02 ± 0.86 ^BC^ [1.88–5.1]
DS1 = 80 % FC	3.17 ± 0.2 ^d^ [2.81–3.34]	4.29 ± 0.16 ^b^ [4.1–4.48]	5.02 ± 0.06^a^ [4.9–5.07]	4.16 ± 0.79^A^ [2.81–5.07]
DS2 = 40 % FC	3.1 ± 0.13 ^d^ [2.95–3.28]	4.29 ± 0.16 ^b^ [4.02–4.48]	4.84 ± 0.16^a^ [4.69–5.05]	4.08 ± 0.75 ^AB^ [2.95–5.05]
DS3 = 20 % FC	3.27 ± 0.08 ^d^ [3.12–3.38]	3.75 ± 0.08^c^ [3.64–3.86]	4.74 ± 0.17^a^ [4.55–5.03]	3.92 ± 0.64 ^C^ [3.12–5.03]
All drought stress levels combined	3.14 ± 0.25 ^γ^ [1.88–3.38]	4.11 ± 0.27 ^β^ [3.64–4.48]	4.89 ± 0.17 ^α^ [4.55–5.1]	
**Total CaCO**_**3**_**(%)**				
DS0 = 100 % FC	2.54 ± 0.2^f^ [2.23–2.9]	2.73 ± 0.23 ^cdef^ [2.33–2.99]	3.58 ± 0.44 ^ab^ [3–4.12]	2.95 ± 0.55 ^B^ [2.23–4.12]
DS1 = 80 % FC	2.59 ± 0.3 ^ef^ [2–2.9]	3.12 ± 0.44 ^bcde^ [2.6–3.9]	3.2 ± 0.17 ^bcd^ [2.86–3.5]	2.97 ± 0.42 ^B^ [2–3.9]
DS2 = 40 % FC	2.74 ± 0.24 ^cdef^ [2.21–3]	3.49 ± 0.45 ^ab^ [2.9–3.94]	3.95 ± 0.25^a^ [3.7–4.5]	3.39 ± 0.6^A^ [2.21–4.5]
DS3 = 20 % FC	2.68 ± 0.29 ^def^ [2.11–2.99]	3.22 ± 0.27 ^bc^ [2.9–3.62]	3.95 ± 0.55^a^ [3.16–4.6]	3.29 ± 0.65^A^ [2.11–4.6]
All drought stress levels combined	2.64 ± 0.26 ^γ^ [2–3]	3.14 ± 0.44 ^β^ [2.33–3.94]	3.67 ± 0.48 ^α^ [2.86–4.6]	
**Active CaCO**_**3**_**(%)**				
DS0 = 100 % FC	13.74 ± 2.17 ^b^ [10.9–17.05]	9.55 ± 1.7^c^ [7–12.05]	13.98 ± 1.96 ^b^ [11.37–16]	12.42 ± 2.79^A^ [7–17.05]
DS1 = 80 % FC	15.63 ± 3.11 ^ab^ [11.3–20.4]	8.22 ± 1.78^c^ [6‒11]	11.89 ± 0.99 ^bc^ [11.37–14.3]	11.91 ± 3.71^A^ [6–20.4]
DS2 = 40 % FC	18.67 ± 3.27^a^ [13.7–25]	8.66 ± 0.85^c^ [8‒10]	13.66 ± 2.85 ^b^ [10–18.5]	13.66 ± 4.83^A^ [8‒25]
DS3 = 20 % FC	14.74 ± 5.53 ^ab^ [11–25]	8.68 ± 1.05^c^ [7.95–10.4]	13.62 ± 1.7 ^b^ [11.37–16]	12.35 ± 4.22^A^ [7.95–25]
All drought stress levels combined	15.69 ± 4.03 ^α^ [10.9–25]	8.78 ± 1.43 ^γ^ [6–12.05]	13.29 ± 2.08 ^β^ [10–18.5]

**Table 3:** Generalized linear mixed-effects models (GLMM) testing the variation of soil physicochemical parameters according to the effects of biochar doses and drought stress levels.

Variables	*df*	*χ*^2^	*p*-value	Sig.	*χ*^2^	*p*-value	Sig.
		Soil moisture			pH		
Biochar (BC)	2	8.03	0.018	*	49.98	<0.001	***
Drought stress (DS)	3	8.69	0.034	*	2.55	0.466	NS
BC × DS	6	19.24	0.004	**	3.91	0.689	NS
		Electrical conductivity			Soil organic matter		
Biochar (BC)	2	17.77	<0.001	***	1587.51	<0.001	***
Drought stress (DS)	3	45.58	<0.001	***	23.03	<0.001	***
BC × DS	6	155.28	<0.001	***	46.96	<0.001	***
		Total CaCO_3_			Active CaCO_3_		
Biochar (BC)	2	167.37	<0.001	***	135.66	<0.001	***
Drought stress (DS)	3	35.25	<0.001	***	7.00	0.072	NS
BC × DS	6	20.09	0.003	**	16.64	0.011	*

(*df*: degrees of freedom, *χ*^2^: Chi-squared statistics, *Sig*.: statistical significance, ***: *p <* 0.001, **: *p <* 0.01, *: *p* < 0.05, ^NS^: *p* > 0.05).

#### 3.1.2 pH

Biochar was the sole factor that had a highly significant effect (GLMM: *p* < 0.001) on soil pH (Table 3). Conversely, both DS and the DS × biochar interaction did not demonstrate any significant effects (*p* > 0.05) on the change of this parameter. It becomes evident that the application of 5 g/kg of soil of this biochar fertilizer induced an extreme alkalinity, resulting in an average pH value of 8.18. Consequently, the obtained results were categorized into three groups: B1 > B2 > B0 (Table 2).

#### 3.1.3 Electrical conductivity (EC)

The analysis revealed highly significant effects on EC due to biochar doses (GLMM: *χ*^2^ = 17.77, *p* < 0.001), DS (*χ*^2^ = 45.58, *p* < 0.001), and their interaction, DS × biochar (*χ*^2^ = 155.28, *p* < 0.001) (Table 3). Levels of EC exhibited a linear increase corresponding to the application of higher biochar doses, with the highest value recorded at 1369 ± 316 μS/cm for dose B2. Additionally, under the influence of applied DS, EC displayed an increase, reaching 1431 ± 316 μS/cm at DS1 (80 % FC), while the most severe DS level showed a lower value of 1123 ± 458 μS/cm. Furthermore, in the case of the interaction effect, similar to humidity, EC displayed an extreme average of 1998 μS/cm. This was observed with the B1 dose under the condition of DS at 80 % FC (Table 2).

#### 3.1.4 Soil organic matter (SOM)

The accumulation of SOM exhibited a positive and significant evolution under the influence of biochar (GLMM: *χ*^2^ = 1587.5, *p* < 0.001), in contrast to its response to DS alone (*χ*^2^ = 23.03, *p* < 0.001). Additionally, the interaction between DS and biochar was also found to be significant (*p* < 0.001) (Table 3). As the application of biochar doses increased, the accumulation of SOM showed a corresponding increase. SOM content ranged from 1.88 ± 0.25 % for the control soil to 4.89 ± 0.17 % for the highest dose, B2. Conversely, when subjected to DS, SOM declined from 4.02 ± 0.86 % at the 100 % irrigation level to 3.92 ± 0.64 %. The combined effect of DS and biochar revealed that the highest SOM content, reaching 5.1 %, was obtained in this trial under the conditions of 100 % irrigation and the highest biochar dose, B2 (Table 2).

#### 3.1.5 Total CaCO_3 (TCCE)_

The GLMM revealed highly significant effects for both the biochar (*χ*^2^ = 167.37, *p* < 0.001) and DS factors (*χ*^2^ = 35.25, *p* < 0.001), as well as their interaction, DS × biochar (*χ*^2^ = 20.09, *p* = 0.003) (Table 3). The TCCE content exhibited a consistent linear increase with the application of biochar doses, ranging from 2.64 ± 0.26 % for the control soil to 3.67 ± 0.48 % with B2. Similarly, results observed under DS conditions followed the same trend, showing an increase from the lowest value of 2.95 ± 0.55 % at the 100 % FC to 3.29 ± 0.65 % for the most severe water stress (DS3). Notably, the combination of DS3 and B2 facilitated the highest accumulation of TCCE, resulting in the highest average content of 4.6 %. Conversely, the B0—DS1 soil recorded the lowest average value at 2 % (Table 2).

#### 3.1.6 Active CaCO_3_ (ACCE)

The statistical analysis revealed a significant effect for biochar (GLMM: *χ*^2^ = 135.66, *p* < 0.001) and the interaction effect DS × biochar (*χ*^2^ = 16.64, *p* = 0.011). However, the applied DS did not show a significant effect (*p* > 0.05) (Table 3). The ACCE content exhibited a negative trend with increasing biochar doses, shifting from 15.69 ± 4.03 % for the control soil to 13.29 ± 2.08 % for the B2 dose. When DS was introduced alongside the biochar fertilizer, the lowest value, 10 %, was detected as the minimum content. This occurred with the 40 % water level and the biochar dose B1 (Table 2).

### 3.2 Correlations between soil parameters

The correlation matrix revealed significant and positive correlations in the soil, with pH being positively correlated with organic matter (*r* = 0.38, *p* < 0.001), TCCE (*r* = 0.29, *p* = 0.002), and moisture (*r* = 0.23, *p* = 0.015), but negatively correlated with ACCE (*r* = −0.50, *p* < 0.001). Additionally, EC exhibited significant and positive correlations with SOM (*r* = 0.20, *p* = 0.037) and TCCE (*r* = 0.19, *p* = 0.048). Regarding SOM, it was significantly and positively correlated with TCCE (*r* = 0.69, *p* < 0.001), but negatively correlated with ACCE (*r* = −0.29, *p* = 0.003) (Table 4).

**Table 4:** Pearson correlation matrix between morpho-physiological parameters of the plant under biochar amendment and drought stress. Values reported above the diagonal are Pearson correlation coefficients, and those below the diagonal are *p*-values. Significant correlations (*p* < 0.05) are indicated in boldface font.

*P*-values	Pearson correlation coefficients					
	SM	pH	EC	SOM	TCCE	ACCE
Soil moisture (SM)		**0.23**	0.03	0.16	0.13	−0.15
Potential hydrogen (pH)	**0.015**		−0.09	**0.38**	**0.29**	**−0.50**
Electrical conductivity (EC)	0.791	0.355		**0.20**	**0.19**	−0.14
Soil organic matter (SOM)	0.095	**<0.001**	**0.037**		**0.69**	**−0.29**
Total CaCO_3_ (TCCE)	0.179	**0.002**	**0.048**	**<0.001**		−0.10
Active CaCO_3_ (ACCE)	0.132	**<0.001**	0.137	**0.003**	0.316	

### 3.3 The performance of the plant

#### 3.3.1 Morphological evolution of the plant

**Fig. 1:**
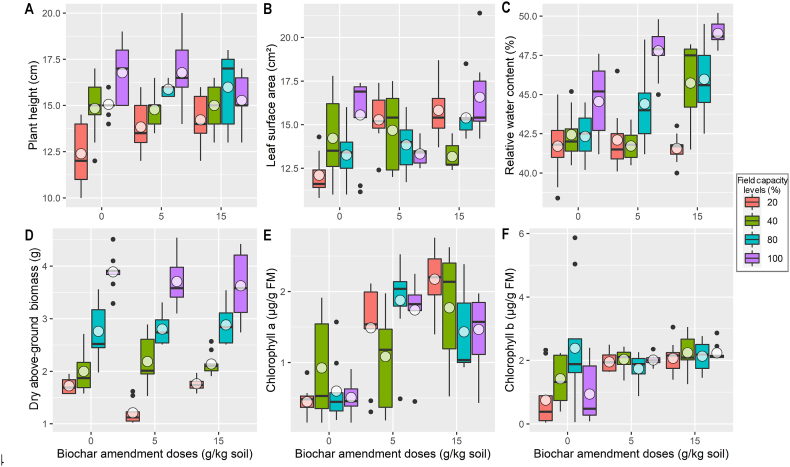
Boxplots representing the variation of wheat plant parameters for different biochar amendments and drought stress treatments (100 %, 80 %, 40 %, 20 % FC). Black solid circles are outliers. (A: plant height, B: leaf surface area, C: relative water content, D: dry above-ground biomass, E: chlorophyll *a*, F: chlorophyll *b*).

Biochar had no significant effect (*p* < 0.05) on plant height and dry above-ground biomass (Table 5). However, the application of DS played a crucial role in determining these two parameters. The GLMMs indicated highly significant effects (*p* < 0.001) for plant height (*χ*^2^ = 65.77) and AGB (*χ*^2^ = 544.79) in response to DS alone (Table 5). When considering the combination of DS and biochar, the GLMM statistics still showed significant effects for both height (*χ*^2^ = 16.56, *p* = 0.011) and AGB (*χ*^2^ = 14.93, *p* = 0.021). Under the influence of DS, plant AGB decreased from 3.74 ± 0.48 g/plant to 1.57 ± 0.31 g/plant, while height also decreased from 16.28 ± 1.68 cm to 13.48 ± 1.60 cm (Fig. 1). The results indicated that when DS was combined with biochar, the AGB decreased further. The highest AGB, 4.54 g/plant, was observed with the B1—DS0 combination. Similarly, the tallest plants, reaching 20 cm, were also found with the B1—DS0 combination, while this value decreased to 12 cm with B1—DS3.

**Table 5:** Generalized linear mixed-effects models (GLMM) testing the variation of plant wheat parameters following the combined effects of biochar doses and drought stress levels under controlled conditions.

Variables	*df*	*χ*^2^	*p*-value	Sig.	*χ*^2^	*p*-value	Sig.
		Plant height			Leaf surface area		
Biochar (BC)	2	3.20	0.202	NS	13.66	0.001	**
Drought stress (DS)	3	65.77	<0.001	***	7.08	0.069	NS
BC × DS	6	16.56	0.011	*	40.10	<0.001	***
		Relative water content			Dry above-ground biomass		
Biochar (BC)	2	44.76	<0.001	***	2.74	0.254	NS
Drought stress (DS)	3	127.96	<0.001	***	554.79	<0.001	***
BC × DS	6	30.44	<0.001	***	14.93	0.021	*
		Chlorophyll *a*			Chlorophyll *b*		
Biochar (BC)	2	83.63	<0.001	***	19.13	<0.001	***
Drought stress (DS)	3	0.93	0.819	NS	5.94	0.115	NS
BC × DS	6	24.78	<0.001	***	18.16	0.006	**

(*df*: degrees of freedom, *χ*^2^: Chi-squared statistics, *Sig*.: statistical significance, ***: *p* < 0.001, **: *p* < 0.01, *: *p* < 0.05, ^NS^: *p* > 0.05).

Regarding the development of leaf area, the GLMM indicated that DS had a non-significant effect (Table 5). leaf surface area (LSA) values only varied between 15.15 ± 2.35 cm^2^ for DS0 (100 % FC) and 14.40 ± 2.12 cm^2^ for DS3 = 20 % FC. In contrast, biochar induced a significant increase (*χ*^2^ = 13.66, *p* = 0.001) in LSA, which increased from 13.78 ± 2.28 cm^2^ to 15.24 ± 1.95 cm^2^ (Fig. 1). Furthermore, the interaction between biochar and DS also significantly improved LSA (*χ*^2^ = 40.10, *p* < 0.001). The maximum LSA of 21.40 cm^2^ was observed with biochar dose B2 and a water level of 100 % FC, while the minimum value of 12 cm^2^ was recorded for the B1—DS2 combination.

#### 3.3.2 Evolution of plant physiological variables

The effect of DS on the content of chlorophyll *a and b* was not significant (GLMM: *p* > 0.05). However, the application of biochar significantly improved the content of both pigments, Chl *a* (*χ*^2^ = 83.63, *p* < 0.001) and Chl *b* (*χ*^2^ = 19.13, *p* < 0.001) (Table 5). For chlorophyll *a*, the content was 0.62 ± 0.46 μg/g DM for the control and 1.71 ± 0.65 μg/g DM for the B2 dose. Chlorophyll *b* ranged from the lowest level of 1.37 ± 1.35 μg/g DM for control plants to 2.17 ± 0.45 μg/g DM for plants amended with biochar dose B2 (Fig. 1). The results for the DS × biochar interaction indicated a highly significant effect (*p* < 0.001) for chlorophyll *a and a* significant effect (*p* = 0.006) for chlorophyll *b* (Table 5). Chlorophyll *a* contents increased from 1.57 μg/g DM for the B0—DS1 level to 2.76 μg/g DM for the B2—DS3 level. Similarly, for chlorophyll *b*, there was an increase in its content from 2.26 μg/g DM for the B1—DS1 level to 3.05 μg/g DM for B2—DS3.

The statistical analysis for RWC showed a significant biochar effect (GLMM: *χ*^2^ = 44.76, *p* < 0.001), DS effect (*χ*^2^ = 127.96, *p* < 0.001), and DS × biochar interaction (*χ*^2^ = 30.44, *p* < 0.001) (Table 5). RWC improved from 42.76 ± 2.12 % for the B0 dose to 45.54 ± 3.20 % for B2. However, there was a decrease with DS, from 47.08 ± 2.50 % for DS0 to 41.97 ± 1.67 % with DS3. Concerning the interaction between biochar and DS, an increase was observed with the combined increase of biochar doses and DS. With the B0—DS1 level, a content of 44.50 % was observed, and this content increased to 48.50 % for B1—DS1, reaching a maximum of 49.50 % with the B2—DS1 level (Fig. 1).

### 3.4 Correlations between wheat plant variables

**Fig. 2:**
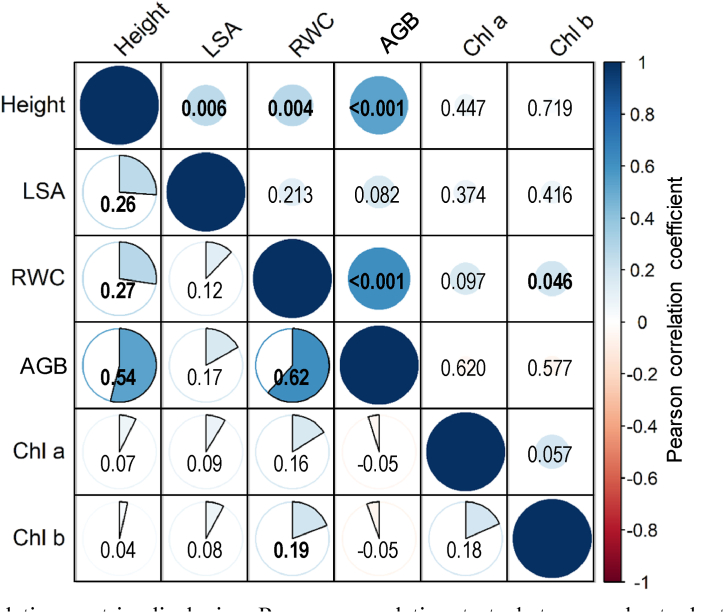
Correlation matrix displaying Pearson correlation tests between wheat plant parameters measured under different biochar amendments applied for tree drought stress levels. Values of correlation coefficient are mapped to color and represented in circle charts (above the diagonal), whereas the *p*-values of the test are shown below the diagonal. Significant correlations (*p <* 0.05) are expressed in bold font. Abbreviations: (AGB: dry above-ground biomass, Chl a: chlorophyll *a*, Chl b: chlorophyll *b*, Height: plant height, LSA: leaf surface area, RWC: relative water content).

The correlation matrix highlighted three significant correlations for plant height (Fig. 2). The first one was with LSA (*r* = 0.26, *p* = 0.006), the second correlation was recorded with the RWC (*r* = 0.27, *p* = 0.004), and then a correlation between the height and the AGB (*r* = 0.54, *p* < 0.001). We also noted a correlation between AGB and RWC (*r* = 0.62, *p* < 0.001), and also between RWC and chlorophyll *b* (*r* = 0.19, *p* = 0.046).

### 3.5 Relationships between soil properties and plant traits under biochar applications

**Fig. 3:**
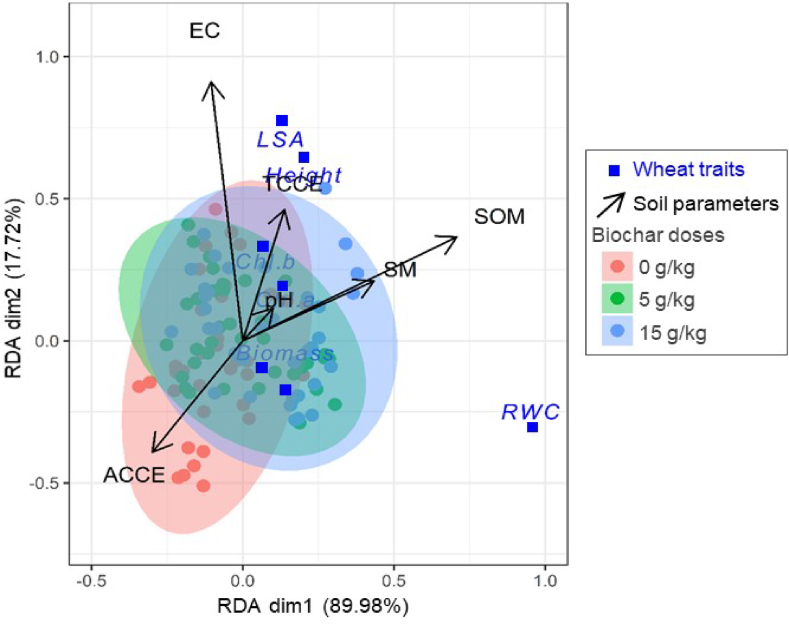
RDA tri-plot diagram of the axes 1 and 2 of the redundancy analysis showing the relationships between soil characteristics and wheat plant parameters under three doses of biochar. Abbreviations: (ACCE: active CaCO_3_, Biomass: dry above-ground biomass "AGB", Chl.a: chlorophyll *a*, Chl.b: chlorophyll *b*, EC: electrical conductivity, Height: plant height, LSA: leaf surface area, pH: potential hydrogen, RWC: relative water content, SM: soil moisture, SOM: soil organic matter, TCCE: total CaCO_3_).

Contrary to EC, the RDA test indicated that the contribution of biochar led to an increase in SOM and SM, an improvement that was positively correlated with the RWC of the leaves. It appears that, unlike the B2 dose, the B1 dose favored better improvement in SM (Fig. 3); however, the inverse relationship between TCCE and ACCE showed that the accumulation of TCCE under the effect of the applied biochar doses limited the formation of ACCE. According to the RDA test, it was detected that the high content of TCCE in the soil limits the development of the wheat plant, and the application of biochar in the soil did not result in an increase of plant AGB and height. However, the effect of this biofertilizer highlighted a physiological improvement, as evidenced by the increase in the content of photosynthetic pigments.

## 4 Discussion

The increase in SM confirms the positive role of biochar in improving SM content. Biochar is rich in organic components; its application improves the porosity, facilitating the improvement of water retention in the soil [[28], [29], [30]]. Also, it appears that as the levels of DS increase, the water content in the soil weakens, a logical observation, as the water supply to the soil is decreased, leading to decreased SM. However, this decrease is less accentuated when the application of biochar is combined with DS, which is explained by the role of this fertilizer in saving water in its interstices [31], thus inducing a maintenance of SM. The humidity of the pots treated with the B1 dose exceeds that of B2, this observation is explained by the high amount of organic matter obtained with the B2 dose compared to B1, so with this dose of biochar (B2), the organic matter plays the role of a sponge by retaining the water through adsorption effect [32], thus limiting its passage into the soil. In this respect, the RDA showed a strong positive correlation between organic matter and SM, a correlation that is more decisive especially with the B2 dose. Our results corroborate with [33,34] who report an increase in hydraulic conductivity and soil water retention following a biochar amendment. On their side, Kim et al. [35] demonstrated an increase in the order of 102 % and 70.2 %, respectively, with the dose of 10 t/ha and 30 t/ha of biochar.

The observed positive correlation in this trial between pH and TCCE elucidates the alkalizing impact of biochar on this parameter. The analyses conducted on this biofertilizer reveal a significant TCCE content, which accounts for the rise in pH. In their respective studies, authors [36,37] have noted that biochar contains varying concentrations of alkaline ashes that contribute to soil alkalinity. Interestingly, this alkalizing effect diminishes with higher biochar doses, a trend consistent with findings from several researchers [[38], [39], [40]] who have observed that excessive biochar application at very high doses can marginally lower soil pH. In general, it becomes apparent that soil treated with biochar tends to exhibit increased electrical conductivity (EC). This EC rise can be attributed to the presence of diverse organic and inorganic minerals within the fertilizer [41].

The composition of this biosolid is rich in various elements, including hydroxides and oxides of Ca^2+^, Mg^2+^, K^+^, and Na^+^. Upon addition to soils, these elements contribute to an increase in EC [42]. In this trial, the observed increase in EC under DS aligns with existing literature [43]. Under DS conditions, soil acts as a reservoir for various salts, both anions and cations, raising its osmotic potential to counteract evaporation, resulting in an EC increase [42,44]. Furthermore, the combined effect of biochar and DS leads to higher EC compared to the control soil. The correlation between soil organic matter and EC suggests that the dissolution of diverse minerals from biochar into the soil organic matter contributes to this effect [45]. Indeed, several studies indicate that this fertilizer serves as a source of various mineral salts, leading to an EC increase when incorporated into the soil. Notably, the B1 dose releases salts more prominently into the soil, resulting in higher conductivity than the B2 dose. This can be attributed to the higher organic matter content in B2, which restricts the release of salts into the soil solution. As organic matter in the soil increases, it enhances the adsorption and fixation of salts, reducing their release into the soil solution [8]. Additionally, similar to moisture content, the highest moisture values are observed with the B1 dose. It is likely that this high moisture content promotes the dilution of mineral matter from the biochar, thereby contributing to the rise in EC.

As previously mentioned, the presence of biochar has a positive impact on soil organic matter content. Zaheer et al. [46] reported that adding 38 g/kg of biochar to soil increased organic matter content by approximately 11.86 %. The biochar utilized in this experiment is produced through pyrolysis, involving the high-temperature heating of tree trunks. The introduction of this pyrolyzed carbon into the soil enhances its organic matter reservoir [28,47]. Several studies have indicated that pyrolyzed carbon is readily soluble, and consequently, with improved soil moisture, a significant portion of organic matter from the biochar dissolves in the soil [48]. In contrast to our findings, Goranov et al. [49] suggested that freshly prepared biochar contains negligible amounts of pyrolyzed carbon.

The application of DS hampers the mineralization of organic matter. However, with higher biochar doses, the soil retains moisture for longer periods [50], facilitating efficient enzymatic activities and subsequently enhancing organic matter content [51]. Adequate soil moisture is crucial not only for plant growth but also for stimulating various humification processes within the soil [52]. It should be noted that biochar promotes an increase in the soil microbial population, intensifying its activity [53]. In this regard, the study [54] reported a positive effect of biochar on the decomposition of pre-existing organic matter in the soil. Mansoor et al. [55] reported that biochar application enhances soil fertility, improving water and mineral uptake.

The results obtained for TCCE in this study indicate that the soil has a low limestone content [56]. The addition of biochar leads to an elevation in TCCE. Similar to pH, the CaCO_3_ content in the biochar contributes to the overall TCCE in the soil. When biochar amendment is combined with DS, there is an increase in the accumulation of TCCE. The water deficiency in the soil does not promote the conversion of TCCE into active calcium carbonate equivalent (ACCE), resulting in its accumulation in the soil. However, in contrast to TCCE, ACCE content decreases compared to the control soil. Without biochar, the ACCE content is relatively high (between 10 % and 20 %) [57]. It is evident that the presence of biochar reduces this content. This decrease can be attributed to the negative correlation observed between pH and active calcium carbonate equivalent (ACCE). Therefore, as pH becomes more alkaline, the formation of ACCE from total calcium carbonate equivalent (TCCE) is reduced [56]. Notably, this decrease is particularly pronounced at the B1 treatment level, which corresponds to the highest pH recorded in this experiment. It is worth noting that the negative correlation identified between ACCE and organic matter indicates that TCCE acts as a protective matrix, with its fine particles shielding residual organic matter from degradation by soil microorganisms. This protective effect restricts the transformation of organic matter into ACCE, resulting in lower ACCE levels [[11], [39]]. Furthermore, the interaction between DS and biochar also contributes to the limitation of ACCE formation. Additionally, as per the results obtained in this study, the soil in all pots receiving biochar exhibits increased salinity [45]. This rise in electrical conductivity (EC) is attributed to the release of various organic and inorganic minerals from the biochar [58], combined with an increase in osmotic potential in the soil due to DS. These combined effects do not promote the biological activity of soil microorganisms and further inhibit the conversion of TCCE into ACCE [59].

Plant AGB and height both decrease under DS, a common response adopted by plants to reduce their water requirements when water availability is limited [8,60]. The positive correlations observed between AGB and the water content of plants indicate that as water content decreases in the plant, AGB production also decreases. Similarly, the correlation between height and AGB shows that reduced plant height limits the amount of biomass produced. These findings align with previous studies [61], which reported reduced plant height in wheat subjected to drought during critical growth stages. Additionally, Zhao et al. [62] observed a reduction in plant height in *Brassica napus* under water deficit conditions.

In terms of wheat production, both AGB and stubble height were not significantly improved by biochar in this trial. It is possible that the fertilizer did not have a substantial impact on plant growth during the relatively short duration of the greenhouse trial, which lasted four and a half months. It is worth noting that some studies have reported that the initial months following biochar application may not show immediate improvements in plant development; these effects may become more evident over time [42,63]. In our case, the highest biochar dose applied was 15 g/kg of soil, which could have contributed to limited growth. This observation is consistent with previous research [64], which suggests that doses exceeding 10 T/Ha of biochar may reduce plant growth. Furthermore, some studies have reported varying impacts of biochar on plant size and root length [58]. In contrast, Agegnehu et al. [[65], [66]] found that biochar application on rice plants under stress conditions increased leaf area and plant height. Overall, biochar can enhance wheat growth and yield-related metrics, such as plant height, fertile tiller count, and grain yield [46].

Unlike AGB and height, biochar demonstrated a positive effect on leaf development. This improvement can be attributed to the role of biochar in providing essential nutrients, particularly nitrogen [[1], [19], [66]]. Analysis of the biochar used in this study revealed a high proportion of nitrates (Table 1), a crucial element for leaf development [67,68]. Several studies have highlighted biochar's ability to act as an "N-trap" in the soil, enhancing the plant's utilization of nitrogen [69]. Notably, our trial results indicate that leaf area increases with higher biochar application rates and moisture levels. This effect can be explained by the positive impact of moisture on the efficient dissolution and release of nutrients from biochar, thereby promoting robust leaf development.

The chlorophyll pigments (*a* and *b*) also increase with biochar application, and the combined effect of DS and biochar further enhances the formation of these pigments. Biochar serves as an important source of various essential elements needed for chlorophyll composition, including nitrogen and magnesium [70]. These elements accumulate in response to both DS and biochar application rates, leading to improved plant nutrition and enhanced photosynthetic activity [71]. These findings are consistent with a previous study [18], which reported increased chlorophyll pigment content in tomato plants under the influence of biochar, even under severe drought conditions. Furthermore, the association of DS and biochar led to improved leaf water content, benefiting the synthesis of chlorophyll *b*. The positive correlation between RWC and chlorophyll *b* confirms this effect. Abideen et al. [72] observed increased chlorophyll pigment concentrations when using biochar and manure, with values of 297 % and 294 %, respectively, compared to control plants. Under water deficit conditions, biochar treatment increased chlorophyll *a*, chlorophyll *b*, and total chlorophyll levels by 18 %, 48 %, and 35 %, respectively, compared to the control [73].

Biochar application has been revealed to enhance the defense mechanisms of plant leaves during drought conditions by increasing the activity of protective enzymes and electron transfer in crops. This, in turn, helps minimize the damaging effects of drought stress on the photosynthetic apparatus [74]. Drought stress typically leads to a significant reduction in chlorophyll content, as noted by Gharred et al. [75]. However, biochar application has been shown to significantly increase chlorophyll content, both under low and high soil water potential conditions. Jabborova et al. [76] also found that the use of biochar, particularly under salt stress and drought conditions, increased the concentration of chlorophyll *a, b*, and carotenoids in maize leaves.

Furthermore, according to Jabborova et al. [4], adding biochar significantly increases the chlorophyll, nitrogen (N), and carbon (C) content of soybean plants in conditions resembling drought. Numerous studies [1,77] have reported that biochar can enhance the activity of protective enzymes and antioxidants in plants, which can mitigate the negative effects of drought stress on photosynthesis. Additionally, biochar has the potential to improve stomatal conductance and water use efficiency, ultimately increasing the rate of photosynthesis [78]. Overall, the mechanisms through which biochar promotes photosynthesis under drought stress are likely complex and multifaceted, and may vary depending on the specific plant species and environmental conditions. Nevertheless, research results suggest that improving soil properties, enhancing antioxidant activity, and increasing chlorophyll content are among the key mechanisms by which biochar can enhance photosynthesis under drought stress.

It is important to note that water reserves in leaves tend to decrease under drought stress due to the dehydration of plant cells [79]. Our results align with those of previous studies [58], which have reported a loss in leaf water content under drought conditions. However, the application of biochar helps preserve leaf water content, likely due to its positive impact on soil moisture retention. Biochar can enhance soil characteristics such as water holding capacity and soil aeration, thus increasing water availability to plants [46,78,80,81]. This trend is further confirmed by the Redundancy Analysis (RDA), which demonstrates a positive correlation between improved soil organic matter and soil moisture content with relative water content (RWC) in plant leaves. In the face of the substantial salt load introduced under the combined influence of DS and minerals from biochar, plant cells adjust their osmotic potential by accumulating more solutes in their vacuoles. This adaptation allows them to limit water loss and maintain turgidity, positively impacting the stability of chlorophyll pigments [82].

## 5 Conclusion

In conclusion, the incorporation of biochar into soil has demonstrated its potential to enhance soil fertility and alleviate the adverse impacts of drought stress on plant growth and photosynthetic processes. Biochar effectively improves soil water retention, which subsequently benefits plant water status and promotes the accumulation of chlorophyll pigments, leading to increased photosynthetic activity and better leaf area development. This positive effect is primarily attributed to the organic matter content within biochar, enriching the soil and enhancing its overall fertility. However, it is crucial to note that the effectiveness of biochar application can vary depending on the dosage. Our findings suggest that a lower dose of 5 g/kg of biochar appears to yield more favorable results in terms of soil improvement compared to a higher dose of 15 g/kg. Additionally, it is important to consider the potential trade-off associated with biochar application, specifically its impact on soil alkalinity. The increase in soil alkalinity, while an unintended consequence, may offset some of the positive benefits of biochar, particularly in regions with naturally calcareous soils, as often found in arid and semi-arid areas. To further refine our understanding and optimize the use of biochar as a soil amendment, future research should aim to explore a range of biochar dosages to determine the most effective and sustainable application rate. This investigation should prioritize finding a balance that maximizes soil fertility improvement without inducing undesirable effects such as excessive soil alkalinity. By doing so, we can harness the full potential of biochar as a valuable tool for sustainable agriculture in water-limited environments.

## Funding statement

This research did not receive any specific grant from funding agencies in the public, commercial, or not-for-profit sectors.

## Data availability statement

Data will be made available on request.

## CRediT authorship contribution statement

**Sonia Boudjabi:** Conceptualization, Data curation, Investigation, Methodology, Project administration, Resources, Writing – original draft, Writing – review & editing. **Nawal Ababsa:** Conceptualization, Data curation, Investigation, Methodology, Project administration, Resources, Writing – original draft, Writing – review & editing. **Haroun Chenchouni:** Conceptualization, Formal analysis, Validation, Visualization, Writing – original draft, Writing – review & editing.

## Declaration of competing interest

The authors declare that they have no known competing financial interests or personal relationships that could have appeared to influence the work reported in this paper.
